# Recent Advances in Bacteriophage Based Biosensors for Food-Borne Pathogen Detection

**DOI:** 10.3390/s130201763

**Published:** 2013-01-30

**Authors:** Amit Singh, Somayyeh Poshtiban, Stephane Evoy

**Affiliations:** 1 Department of Electrical and Computer Engineering, University of Alberta, Edmonton, AB T6G 2V4, Canada; E-Mails: poshtiba@ualberta.ca (S.P.); sevoy@ualberta.ca (S.E.); 2 National Institute for Nanotechnology, University of Alberta, Edmonton, AB T6G 2M9, Canada

**Keywords:** bacteriophage, biological probes, biosensors, food-borne pathogens, signal transduction, pathogens

## Abstract

Foodborne diseases are a major health concern that can have severe impact on society and can add tremendous financial burden to our health care systems. Rapid early detection of food contamination is therefore relevant for the containment of food-borne pathogens. Conventional pathogen detection methods, such as microbiological and biochemical identification are time-consuming and laborious, while immunological or nucleic acid-based techniques require extensive sample preparation and are not amenable to miniaturization for on-site detection. Biosensors have shown tremendous promise to overcome these limitations and are being aggressively studied to provide rapid, reliable and sensitive detection platforms for such applications. Novel biological recognition elements are studied to improve the selectivity and facilitate integration on the transduction platform for sensitive detection. Bacteriophages are one such unique biological entity that show excellent host selectivity and have been actively used as recognition probes for pathogen detection. This review summarizes the extensive literature search on the application of bacteriophages (and recently their receptor binding proteins) as probes for sensitive and selective detection of foodborne pathogens, and critically outlines their advantages and disadvantages over other recognition elements.

## 1. Motivation for Early Detection

Bacteria are omnipresent and thus their existence in food is natural. While the majority of bacterial strains are harmless or even beneficial to humans, several others, being pathogenic in nature, can cause severe threats to health and safety and consequentially inflict tremendous burden on our socio-economic balance and health care systems. The World Health Organization estimates that some 2.2 million deaths occur annually due to food and water-borne illnesses, and 1.9 million among them are children. The cooking process successfully kills any potential bacteria that are present in food, however, food styles have changed significantly in recent years, and more processed and ready-to-eat packaged foods are available, which increases the chance of exposure to pathogenic contamination. Processed meat, poultry, vegetables and milk products are among the most probable carriers of potent food-borne pathogens, including *E. coli*, *Salmonella*, *Listeria* and *Campylobacter jejuni* and there have been numerous incidents of product recalls across United States in past years. *E. coli* O157:H7 was considered a rare serotype when first reported in 1983, but is now one of the major causes of food-borne diseases in developed countries [1,2]. The infectious dose of these pathogens is very low (∼10 bacteria) and emergence of drug-resistant strains and biological warfare agents has further compounded the problem. Monitoring food has therefore been argued as the most important priority towards national and international health and safety with global emphasis on rapid and early detection of pathogen contamination in food and water.

Conventional pathogen detection methods largely rely on microbiological and biochemical analysis, which are highly accurate but overly time consuming, cost-ineffective and non-amenable to integration for on-site diagnosis. Besides, successful execution of pathogen identification and detection by conventional methods require extensive training and experience. Alternative rapid but accurate methods for pathogen detection have therefore been sought to overcome these limitations. Advances in immunological methods such as enzyme-linked immunosorbent assay (ELISA) have paved the way towards development of easier and quicker pathogen detection methods, relying on the recognition specificity of antibodies (Abs). Immunological methods however suffer from cross-reactivity of polyclonal Abs, high production cost of monoclonal Abs, need for sample pre-processing and pre-enrichment due to low processing sample volume and lower limit of detection. Polymerase chain reaction (PCR) is yet another method that leverages the nucleic acid complementarity-based specificity of pathogen detection. Recently, more sophisticated traditional analytical methods such as liquid/gas chromatography coupled with mass spectrophotometry have been used for more accurate analysis of pathogen. Although these methods have enjoyed tremendous popularity, their feasibility towards point-of-care onsite pathogen monitoring tools is hard to realize. Development of alternative tools for fast, accurate and sensitive detection of pathogens has therefore been an area of continued interest to researchers across the globe.

**Figure 1.**
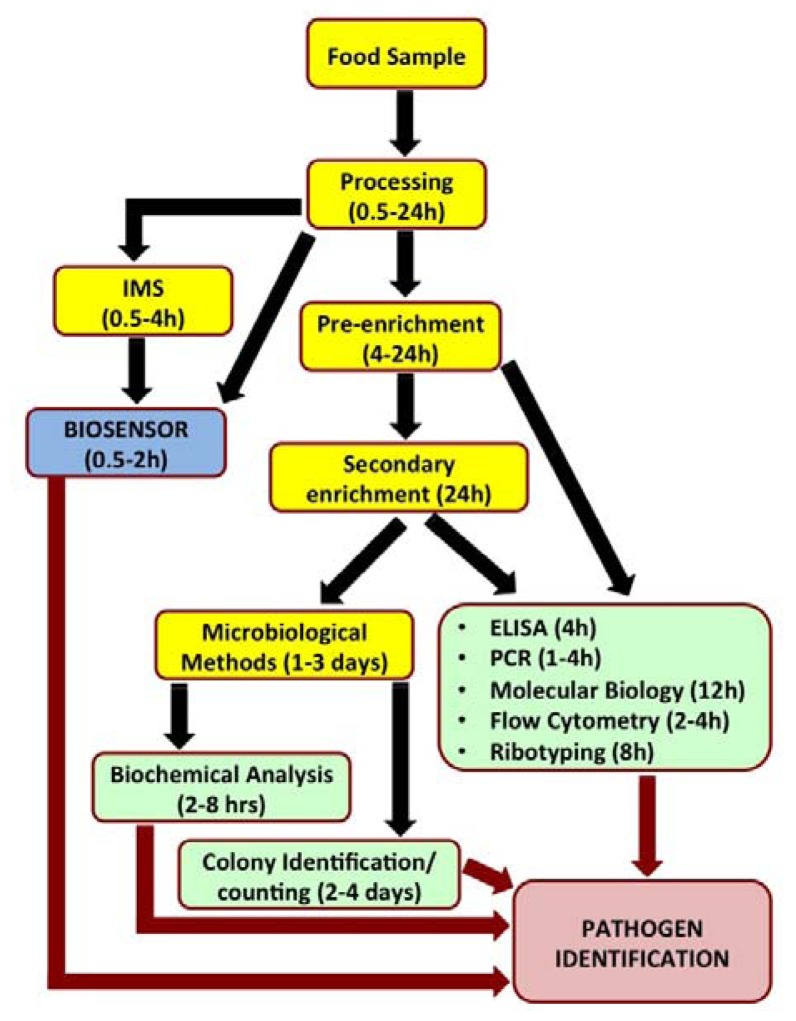
A flowchart elucidating the processing steps involved and relative time taken in detecting a pathogen in a food sample. IMS stands for immune-magnetic separation where particles with magnetic properties are modified with target-specific antibody/antibody fragments for capture and subsequent purification using external magnetic field.

Biosensors have recently been looked upon as attractive alternatives to the existing conventional pathogen detection platforms. Biosensors are analytical devices which translate a specific bio-recognition event into a measurable signal. They offer several advantages such as high degree of sensitivity and specificity of detection, minimal sample preparation, cost-effectiveness, miniaturization and portability for *in situ* real time monitoring and reduced overall time required for detection. Figure 1 outlines the steps involved in analysis of a food sample by various popular detection methods and time involved to reach a conclusive pathogen identity.

**Figure 2.**
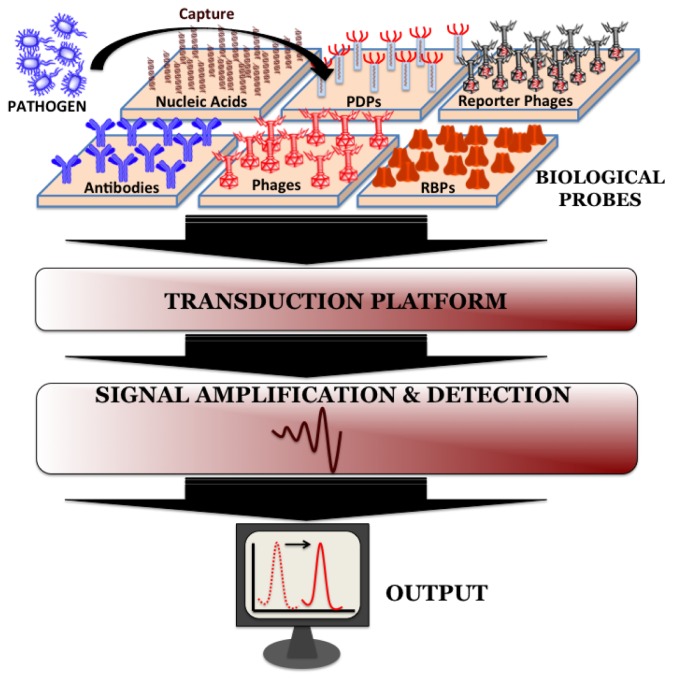
A schematic of various components of a typical biosensor highlighting the available phage-based molecular probes for pathogen detection.

Biosensors can be directly applied for the detection of pathogen in processed food matrices. Such processing methods usually include mincing and homogenization of food samples in the presence of detergents and/or proteolytic enzymes and the choice of processing method depends on the type and complexity of the sample. Biosensors do not require the time-consuming sample pre-enrichment and secondary enrichment steps and therefore can accurately predict the level and kind of food contamination much faster compared to conventional microbiological, immunological and molecular biological methods. A typical biosensor has three associated components: the sensor platform functionalized with a bio-probe to impart specificity of recognition, a transduction platform that generates a measurable signal in the event of analyte capture and the amplifier which amplifies and process the signal to give a quantitative estimate of analyte capture. Figure 2 illustrates the different components of a biosensor. Biosensors for monitoring food and water samples have not yet been commercialized, unlike those available for medical diagnostics, yet the recent developments show tremendous possibility. This review will discuss different bio-probes and transduction platforms that have been successfully leveraged for pathogen detection with a focus on recent advances in biosensor technology for on-site detection.

## 2. Recognition Elements

Bio-probes are often argued as the most vital component of any biosensor since they define the recognition specificity for the pathogen detection. Ideal attributes of any recognition element would be high stability, ease of immobilization on sensor platform and recognition specificity towards host with minimum cross-reactivity from interfering pathogens. The popular bio-probes that have been employed on biosensor surface for pathogen detection are nucleic acids, antibodies, whole phages, phage-display peptides (PDPs) and most recently phage's receptor binding proteins (RBPs).

### 2.1. Nucleic Acids

The fundamental principle behind nucleic acid based detection lies in the sequence complementarity. The careful choice of probe is essential to maintain specificity of detection. Deoxyribonucleic acid (DNA), ribonucleic acid (RNA) and peptide nucleic acids (PNAs) are the molecular probes that have been explored for such applications. The major advantage of using DNA- based probes is the ability to amplify a desired target DNA sequence from the host pathogen using PCR and consequently augment the signal generated by the biosensor in the event of hybridization on the detection platform. RNA could similarly be amplified by reverse transcription PCR (RT-PCR) using RNA polymerase enzymes to similar effect. Alternatively, PNAs are pseudopeptide DNA mimics that show high binding affinity to DNA or RNA by sequence-specific complementary base pairing. They are therefore looked upon as attractive substitutes for DNA- or RNA-based probes due to their improved binding characteristics and better stability against physical, chemical and biological degradation. The use of PNAd as biological probes for pathogen detection is a relatively new but rapidly growing area of investigation but their application is limited by the high cost involved in the synthesis of these probes. DNA and RNA based detection approach therefore is simple, stable, versatile, rapid and cost-effective. Besides, the development of microarray technology [3] and multiplex-PCR [4] provides opportunities for detection of several pathogens simultaneously in complex food matrices. In addition, nucleic acid-based probes (especially DNA) are highly stable in a variety of solvents [5] and buffers, which facilitates their application in a wider range of food samples. The advancement in the nucleic acid-based probe technology has led to several commercialized products for pathogen detection mostly for clinically relevant samples (Table 1). Commercialized nucleic-acid based kits for foodborne pathogen detection are listed in Table 2.

Though nucleic acid hybridization-based detection systems are tremendously popular for pathogen identification, they have several drawbacks that limit their application. PCR-based amplification methods rely heavily on the purity of the template nucleic acid and are therefore prone to contaminations that would amplify and result in false positives. Similarly, degradation of the template nucleic acid could also result in a false negative result. One such product, LCx (Abbott Laboratories, Abbott Park, IL, USA), a ligase chain reaction based system for *Chlamydia* detection was pulled from the market in 2003 due to problems with reproducibility of results [6]. Nucleic Acid Hybridization (NAH) detection systems are also incapable of predicting the viability of the bacteria and thus the true bacterial load in a sample. Besides, these systems cannot be used to detect toxins produced by certain bacteria in a food sample. Despite these limitations, NAH-based systems have enjoyed considerable success and have been extensively studied. A details account of nucleic acid based detection system has been reviewed [7] and is recommended to the readers.

### 2.2. Antibodies (Abs)

Antibodies have been extensively explored as bio-probes for pathogen detection and monitoring due to the ease of their immobilization on biosensor surface and high level of specificity (k_d_ ≈ 10^−7^–10^−11^) towards their target. Polyclonal and monoclonal Abs, Abs fragments, recombinant Abs and llama bodies have been successfully employed for detection of pathogens, their spores as well as toxins. They have also been simultaneously used for immune-magnetic separation of pathogens during sample processing for pre-enrichment and pre-concentration [8]. Enzyme-linked immunosorbent assay (ELISA) is the most commonly used method for Ab-based detection, though they have been successfully integrated on other biosensor platforms as well. The PCR-based target amplification and ELISA-based detection specificity have also been combined as PCR-ELISA to attain improved detection limits. Perelle *et al.* demonstrated that PCR-ELISA could be successfully employed to detect five *Salmonella* cells in milk or meat samples of 25 g size [9]. Abs have similarly been integrated into optical [10], electrochemical [11], mass-based [12], magnetic [13], surface-acoustic wave (SAW) [14] and cantilevers [15] based platform for detection of pathogen mostly in clinical samples though there is a dearth of commercialized systems for analysis of food samples.

Abs as bio-probes however suffer from several drawbacks that limit their application. They are highly prone to physical (temperature, pH), chemical and enzymatic damage. They have to be stored in a controlled refrigerated environment and even then the shelf life is low, limiting their application in *in situ* conditions outside the lab. Polyclonal Abs have several recognition epitopes and thus show cross-reactivity, while monoclonal antibodies, though specific to a single epitope, involve high production costs. Production of the Abs also involves immunization of animals, which poses ethical issues with their application. Stability of the Abs at higher temperature has been addressed to some extent by production of llama bodies, which are truncated Abs with single heavy chain (V_H_) with small antigen binding site and lack light chain (V_L_) [16,17]. These llama bodies have been found to be stable up to 90 °C and their engineered clones have been successfully applied in pathogen detection [18,19]. Phage display Abs is yet another approach that overcomes several shortcomings of conventional Abs, which will be discussed in detailed in the next section. Byrne *et al.* have recently reviewed the principles, problems and potential application of Ab-based sensors for detection of pathogens and toxins [20]. Table 2 provides a list of some commercially available kits for the detection of foodborne pathogens along with the method of detection, company name, product name and limit of detection if available.

### 2.3. Phages

Phages are obligate parasites that lack their own metabolic machinery. They use their bacterial hosts for multiplication and propagation of mature virions. Most phages recognize their host very specifically to the strain level of bacteria, with few exceptions, such as *Listeria* phage A511 that identifies, binds and kills within an entire genus [21] while some phages show inter-species binding capability. Phages bind to their host bacteria, inject their DNA and take over the host machinery to propagate new virions that lyse the bacteria to infect new host (lytic phages) or integrate their genome in to the host DNA, remain dormant until stimulated for replication and propagation (lysogenic phage). With an estimated pool of 10^31^ phages existing in the environment, this provides a unique class of recognition elements that can be exploited not only for bacterial identification and binding on biosensor surface but also as therapeutic biocontrol agents. The following sections will outline a detailed description of phage-based recognition elements that have been employed as bio-probe on sensor platforms for pathogen detection.

#### 2.3.1. Wild-Type Phages

The inherent ability of the phages to bind to their target pathogen has been exploited to design biosensor surfaces using physical and chemical functionalization. The physical functionalization is achieved by surface adsorption, which is simple and straightforward, but gives inconsistent and unstable immobilization density. Physical adsorption nonetheless has been used for detection of *Staphylococcus aureus* using lytic phages (detection limit ∼ 10^4^ cfu·mL^−1^) by surface plasmon resonance (SPR) [22] and *Salmonella* using magnetoelastic sensor in suspension [23] and fat free milk (detection limit ∼ 10^3^ cfu·mL^−1^) [24]. Similarly, physical adsorption of the phages on sugar and amino acid modified gold surfaces [25] as well as surface modified silica particles [26] for pathogen capture have also been reported in the literature. The ELISA-based binding strength study of phages *versus* monoclonal antibody against β-galactosidase in *E. coli* reveals that phages (K_d_ ∼ 21 ± 2 nM) bind to their target with similar or better affinity compared to monoclonal antibody (K_d_ ∼ 26 ± 2 nM) [27]. Despite these successful functionalization reports, strong chemical immobilization of phages on a sensor platform is preferred due to several advantages.

The anchoring of phages by chemical bonds on a biosensor detection platform is pertinent to development of a consistent and stable detection system. The advantage of surface modification and chemically anchored immobilization approach was revealed by a methodical study that demonstrates a 7-fold and 37-fold improvement in the phage density respectively, on cysteamine-modified and gluteraldehyde activated gold substrate compared to that by physical adsorption on bare gold susbstrate [25]. This two-step method was further improved by application of dithiobis(succinimidyl propionate) (DTSP) self-assembled monolayer (SAM) where the thiol group binds to the gold surface while the free succinimidyl interacts with the surface amine groups on the phages [28]. Silane chemistry has similarly been applied for silicon based substrates to facilitate P22 phage immobilization for *Salmonella* capture [29] as well as study of phage receptor-host ligand binding strength using atomic force microscopy [30]. In yet another example, electrochemical oxidation was used to generation of carboxyl group on carbon surface followed by amide coupling of T4 phages for subsequent *E. coli* capture [31].

Purity of the phage suspension is an important criterion to consider for chemical functionalization. Phages are amplified in their host bacterial culture to achieve high titers; and despite repeated centrifugation, the contamination of bacterial protein, lipids and carbohydrates could severely affect the efficiency of immobilization and binding ability of the phages. Phage lysate have therefore been purified by a host of methods such as ultra-high speed centrifugation [25], ultra-filtration [32], poly(ethylene glycol) precipitation-gradient centrifugation [33], chromatofocusing [34] and size exclusion chromatography [35]. An interesting study demonstrates that purified phage lysate can be essentially used to systematically study the binding kinetics of the phages on to an activated surface for chemical immobilization. Study with T4, P22 and NCTC 12673 phages model systems revealed that the phage binding kinetics does not follow the idealized and homogenous Langmuir adsorption isotherm but is governed by heterogenous adsorption closely related to Brouers-Sotolongo isotherm [35]. Such rigorous surface binding studies are extremely important for understanding of phage immobilization on a surface and cannot be realized in the presence of contaminations in the lysate. Wild-type phages have been extensively explored for functionalization of biosensor platforms and subsequent pathogen detection.

Wild-type intact phages however suffer from certain drawbacks that limit their application on biosensor platform. Intact phages are biologically active and thus result into the lysis of the host bacterium upon infection that would lead to loss of signal on a biosensor platform [36]. Besides, some phages show enzymatic activity towards their host bacterial surface receptor. For example, P22 phage shows endorhaminosidase activity towards the O-antigen on the surface of *Salmonella enterica*, which results in binding and subsequent detachment of the bacterium. Sf6 phages targeting *Shigella flexnari* shows similar endorhamnosidase activity [37]. Such enzymatic activity would lead to inconsistent signals on a biosensor platform, leading to changes in detection efficiency. Results also suggest that intact phages bound on sensor platform lose their bacterial binding capability upon drying. This could be explained due to the fact that intact phages collapse on the sensor surface upon drying, and consequently their tail fibers are unavailable to bind to the bacterial host [36]. In addition, intact phages have relatively large sizes, which limits their application as bioreceptors on particular sensor platforms such as in the surface plasmon resonance based sensor where detection signal is distance dependent.

#### 2.3.2. Engineered Phages

The ability to manipulate the genetic material of microorganisms has paved the way for tremendous possibilities of creating novel recognition systems for biosensor applications. The following section will discuss the prospective application of genetically modified phage based recognition elements as bio-probes for pathogen detection.

##### Phage Display Peptides

**Figure 3.**
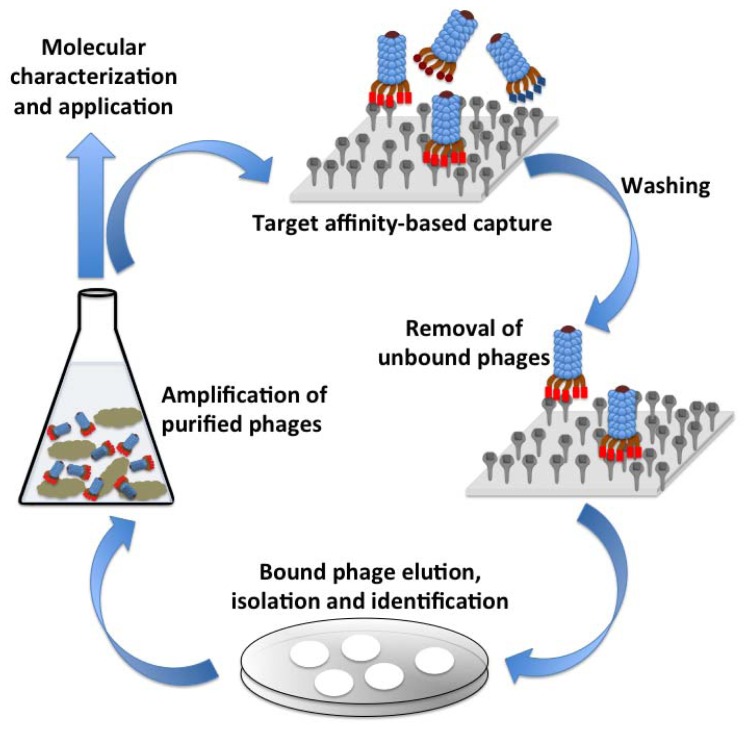
A schematic of affinity-based selection procedure adapted in phage display technology.

Phages have a unique ability to display peptides or proteins on their surface, a technology that was first described in 1985 [38]. This technology enables the screening of proteins or peptide that would have affinity to a variety of target such as carbohydrates, proteins, small molecules or an entire cell. The underlying principal of this method is to fuse the gene encoding for the peptide or protein of interest to the phage surface protein encoding gene(s) resulting in the expression of the hybrid protein on phage surface [39]. Lambda, M13, f1, fd, T4 and T7 phages have most widely been used for the phage display technology. The phage libraries, thus developed can be screened against an immobilized target of interest, the unbound phages are washed away and the tightly bound phages are eluted, propagated and are used as probes against that target. Figure 3 illustrates various events in the phage affinity-based selection for probe development against a target. Peptides, cellular proteins, target specific Abs or Ab fragments [single chain variable fragments (ScFv), antigen binding fragment (Fab), *etc.* have successfully been expressed on the surface of phages for different target and have found application in gene delivery, molecular imaging and developing pathogen detection biosensors [40]. A comprehensive review by Smith and Petrenko summarizes the central paradigm and technical details of the phage display technology [41].

Phage display not only enables peptide- and protein-based acquired recognition specificity towards a pathogen, but has also been exploited to facilitate oriented immobilization of these bio-probes onto a biosensor platform. Gervais *et al.* demonstrated an oriented immobilization of T4 phages using the popular biotin-streptavidin recognition. T4 phages expressing biotin on the head region were immobilized on streptavidin coated gold substrates facilitating exposed tails for specific capture of *E. coli* [42]. In yet another study, biotin carboxyl carrier protein gene (*bccp*) and cellulose binding module gene (*cbm*) were expressed with the small outer capsid protein (*soc*) of T4 phages and the expressed surface ligand was leveraged for the phage immobilization on streptavidin coated magnetic particles and cellulose-based material [43]. Oriented immobilization of phages can also be achieved by a careful study of differences in physical properties in their head region compared to the tail region. Studies with mutant phages reveal that the phage head carry a net negative charge while the tail region has a net positive charge [44]. This charge difference has been used to immobilize *Listeria* and *E. coli* infecting phages on a positively charged cellulose membrane by electrostatic interaction [45]. Such subtle differences in phage properties could be of tremendous importance for successful functionalization of sensor platforms.

##### Reporter Phages

**Figure 4.**
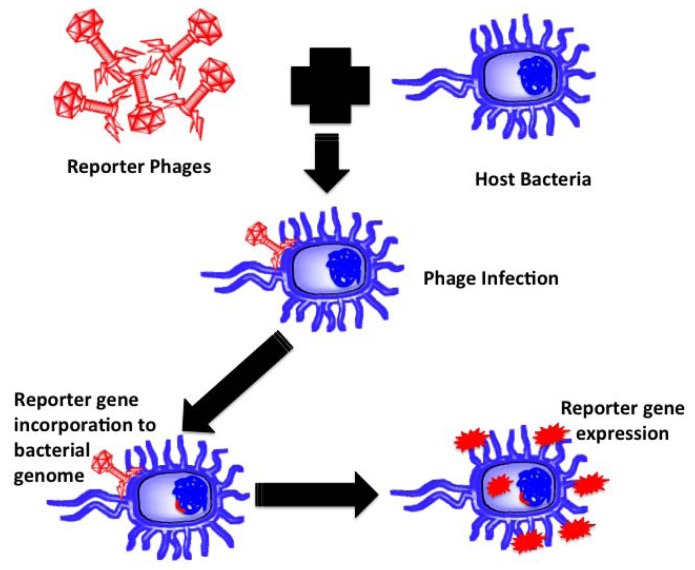
A schematic explaining the underlying principle of reporter phage-based detection of a pathogen of interest.

Reporter phages are genetically modified phages used as a reporting gene carrier, introducing a gene of interest into the host bacteria upon infection. The reporter gene of interest incorporates into the host chromosomes, expressed and codes for a fluorescent or substrate dependent colorimetric marker for subsequent pathogen identification. Bacteriophages, being host dependent for any physiological function, are incapable of expressing the reporter gene by themselves until host infection, thereby confirming the presence of the host bacterium upon gene expression. Prokaryotic and eukaryotic luciferase expressing gene (*lux* and *luc*), *E. coli* β-galactosidase (lacZ) gene, bacterial ice nucleation (*inaW*) gene and green fluorescent protein (gfp) expressing gene have been most commonly used for such applications. The schematic in Figure 4 illustrates the underlying principle of reporter phage technology for subsequent bacterial identification.

Reporter phage-based technology has been successfully leveraged for identification of several pathogens, including *E. coli* [46], *Mycobacterium* [47–49], *Salmonella* [50], *Staphylococcus aureus* [51] and *Listeria monocytogenes* [52]. Loessner *et al.* successfully employed A511::luxAB recombinant phage for detection of one *Listeria* bacterium per gram of sample of artificially spiked ricotta cheese, chocolate pudding and cabbage [53]. Similarly, on more microbiologically complex food samples such as minced meat or soft cheese, 10 bacteria per gram of could be detected using the same system. The sample processing time and pre-enrichment steps were performed in 20 h and the total detection time was estimated to be 24 h compared to four days by conventional microbiological methods [53]. The ability to distinguish between live and dead bacteria is the biggest advantage of reporter phages since the phages will be unable to infect and express the reporter gene in dead bacteria. Reporter phages however suffer from limitations such as phage multiplication inhibition due to prophage presence [54], DNA restriction-modification system [55], presence of specific phage inhibition genes [56] and antiviral bacterial immunity system [57].

##### Phage Receptor Binding Proteins (RBPs)

Some very recent research efforts has led to the evolution of bacteriophage RBPs as novel probes for pathogen detection. The unique host-specific recognition of the tailed phages comes from the RBPs located on the tail fibers and it is the binding of these proteins that trigger the translocation of the phage genetic material into the host [58,59]. The phages RBPs generally recognize unique proteins or carbohydrate (polysaccharide) sequences on the surface of the host bacterium [60]. The recent advancement in genome information, cloning and molecular biology methods has led to the capability of specifically recognizing the genome of interest, cloning, transfecting and over-expressing the protein of interest. These advancements have greatly benefitted the phage-technology and strategies are devised to identify the phage RBPs and their subsequent application in therapy [61] and pathogen detection [62]. Genetically engineered RBPs offer several advantages over the antibody or intact phage-based technology for pathogen detection. Their agglutination ability towards bacterial cells is found to be similar to the monoclonal antibodies against the bacterial lipopolysaccharides [61]. The RBPs also offer better stability against environment factors such as pH and temperature and resistance against gastrointestinal proteases [61]. Their binding affinity can be easily tailored to the requirement and multi-valency can be imparted if desired. Most importantly, suitable tags can be added to the RBPs sequence at appropriate position without altering their binding affinity and such tags can be exploited for oriented surface functionalization of the RBPs on the biosensor platforms.

Singh *et al.* demonstrated the use of cysteine-tagged P22 phage RBPs on gold surface for capture and detection of *Salmonella enterica* serovar Typhimurium [36]. Their results demonstrate that N-teminus Cys tagged proteins capture bacteria efficiently compared to the C-terminus Cys tagged protein due to preferential orientations. Besides, the endorhamnosidase mutant protein shows a 6-fold improvement in bacterial capture compared to the intact P22 phage as well as phage RBPs with endorhamnosidase activity. In yet another report, the *Campylobacter jejuni* binding RBPs (Gp48) from the phage NCTC 12673 were cloned and overexpressed as glutathione-S-transferase fusion protein (GST-Gp48) [63]. These GST-Gp48 proteins could be successfully functionalized on glutathione derivitized gold surface for specific detection of the *C. jejuni* host bacteria by surface plasmon resonance. The versatility of functionalization of the RBPs was further demonstrated by their immobilization on tocyl-activated Dynabeads^®^ M-80 for specific capture of the host bacteria [64]. In an unpublished result, similar application has been validated for capture of *S. flexnari* using wtRBPs as well as endorhamnosidase mutant D399N RBPs. The results clearly indicated that wtRBPs show a low bacterial capture density of 5.71 ± 0.24 bacteria/100 μ^2^ compared to mutant RBPs (capture density ∼11.07 ± 0.62 bacteria/100 μ^2^) [62]. Although the initial experimental results show great promises for phage RBPs as potential probing element for pathogen detection, much work is still needed to achieve a commercial level biosensor.

## 3. Phage-Based Biosensors

Development of biosensors for monitoring food products and water samples is an interesting research topic. The main objective is to develop enhanced detection technologies with high levels of reliability, sensitivity, and selectivity with short assay times. These are critical factors for inspection of food products in industrial firms considering the short shelf time of products and low infection dose of pathogens in food samples. Efforts have been mainly focused on optimizing the biosensor transducer to improve the detection sensitivity. Bacteriophage-based probes have been combined with various analytical methods to provide the specificity of recognition. We will review the biosensor transduction platforms that have leveraged the phage-based probes for specific detection of food borne pathogens. Phage based biosensors have been successfully used for detection of bacteria directly in fresh produce such as milk [24,65], broth [66], fresh tomato [67], and water [68]. Table 3 summarizes various organisms that have been detected with these detection platforms.

### 3.1. Phage-Based Optical Biosensors

Optical biosensors have been widely investigated for bacterial pathogen detection due to their sensitivity, relatively rapid detection, and adaptability to a wide variety of assay conditions. Optical techniques are divided into two main subcategories, labeled and label-free, based on their working principles. The most commonly employed techniques for bacterial detection are surface Plasmon resonance (SPR), fluorescence/phosphorescence spectrometry and bio/chemiluminescence. In the following section, we will focus on optical biosensors that are combined with bacteriophage-based probes for detection of foodborne pathogens.

#### 3.1.1. Surface Plasmon Resonance Sensors

Surface Plasmon Resonance is the oscillation phenomenon that exists at the interface between any two materials. SPR sensors measure the refractive index near the sensor surface that changes as a result of interaction of target analyte in solution with bioreceptors on transducer surface. SPR has been widely used for real time monitoring of biochemical interactions of small analyte such as DNA hybridization, cell-ligand, protein-peptide, and protein-lipid. SPR systems have also been modified to enable the direct label-free detection of larger biomarkers such as bacterial pathogens. Bacteriophages have been immobilized on SPR sensor surface as probes to provide specificity of recognition for detection of bacteria. The successful detection of *S. aureus* [22], *E. coli* K12 [28], *E. coli* O157:H7 and methicillin-resistant *Staphylococcus aureus* (MRSA) [69] have been demonstrated on phage-immobilized SPR methods. The limit of detection was typically in the range of 10^2^–10^3^ cfu·mL^−1^.

Bacteriophage receptor binding proteins have also been used as biorecognition probes on SPR platforms for specific detection of bacteria. For example, Singh *et al.* immobilized genetically engineered tailspike proteins (TSP) from P22 bacteriophage onto the gold-coated SPR plates, and demonstrated a selective real-time detection of *Salmonella* with the sensitivity of 10^3^ cfu·mL^−1^ of bacteria [36]. They also developed a similar detection platform for detection of *Campylobacter jejuni* bacteria by immobilizing the receptor binding protein (RBP) of *Campylobacter* bacteriophage NCTC 12673 on SPR plates [64]. They expressed GP48 RBPs as a glutathione S-transferase-Gp48 (GST-Gp48) fusion protein and used glutathione self-assembled monolayers (GSH SAM) to immobilize onto surface plasmon resonance (SPR) surfaces. They could thus achieve a limit of detection of 10^2^ cfu·mL^−1^ [64].

#### 3.1.2. Bioluminesence Sensors

Bioluminescence assays are sensitive, rapid, and simple techniques for the quantitative detection of bacteria in samples by measuring the level of light emission from intercellular components. The first step of this assay is bacteria cell lysis to release interacellular components, which are then measured using a bioluminescent reaction with luciferase. The major drawback of this technique is the lack of specificity. The lytic phage is used as a recognition probe to detect and lyse the target bacteria. Blasco *et al.* developed an ATP bioluminescence assay for detection of *E. coli* and *Salmonella Newport* using lytic phage as bioprobe and lysis agent [70]. The sensitivity of bioluminescence assay was improved 10- to 100-fold when adenylate kinase (AK) was used as an alternative cell marker, and fewer than 10^4^ cfu·mL^−1^ *E. coli* could be detected in less than 1 h [70]. A similar assay for *Salmonella* was slower and took up to 2 h [70]. Wu *et al.* showed that the amount of released AK form bacterial cells depends on the bacterial type, the growth stage, the phage type, and the infection time [71]. The use of lytic phage as biorecognition probe provides sensitivity and eliminates the need for lengthy conventional microbiological methods and selective media.

#### 3.1.3. Fluorescent Bioassay

In this technique, fluorescence-labeled bacteriophages are used as staining agents for bacteria. The fluorescently stained bacteriophages recognize and bind to their host bacteria. The complex of phage-bacteria is then detected using flow cytometry or epifluorescent filter technique. The average sensitivity reported so far is around 10^2^–10^3^ cfu·mL^−1^ for epifluorescent microscopy and is 10^4^ cfu·mL^−1^ for flow cytometric detection [78–80]. Goodridge *et al.* combined this technique with immunomagnetic separation method, and could detect between 10 to 10^2^ cfu·mL^−1^ *E. coli* O157:H7 in artificially contaminated milk after 10 h enrichment [65] and 10^4^ cfu·mL^−1^ concentration of *E. coli* O157:H7 in broth [66].

Edgar *et al.* [68] and Yim *et al.* [73] further improved the sensitivity of this approach by using fluorescent quantum dots (QD) to tag bacteriophages. QD improves the intensity and stability of fluorescent signal, and improves the sensitivity of detection platforms such flow cytometry and epifluorescence microscopy. The bacteriophage was engineered with biotin binding peptide on the head. The streptavidin coated QDs were allowed to bind strongly to the biotinylated phages. This method enabled detection of as low as 20 *E. coli* cells in 1 mL water sample in 1 h [68].

The fluorescent assays have also been used for detection of bacterial toxins. Goldman *et al.* applied phage display to select a 12-mer peptide that could bind to *staphylococcal enterotoxin B* (SEB), which causes food poisoning [72]. They could detect as low as 1.4 ng of SEB per sample well in a fluorescence-based immunoassay using a fluorescently labeled SEB-binding phages. Array biosensors were also developed based on a similar principle to simultaneously detect *Bacillus globigii*, MS2 phage and SEB [81].

### 3.2. Michromechanical Biosensors

#### 3.2.1. Quartz Crystal Microbalance Biosensors

A quartz crystal microbalance (QCM) is a very sensitive mass sensor with capability for detection of nanogram changes in mass. A QCM sensor is made of a thin piezoelectric plate coated on both sides with two metallic electrodes. The application of an electrical field across the quartz crystal excites the mechanical resonance. The fundamental wavelength (λ) and resonance wavelength (λ = 2d/n) are determined based on the plate thickness d, and thus the corresponding resonant frequency:

(1) $$f_{n}=n.f_{0}=\frac{n.v}{2d}$$

where ν is the sound velocity, and f_0_, f_n_ are the fundamental and the n^th^ overtone resonant frequency, respectively. The adsorption of mass onto the electrode surface shifts the resonance to lower frequencies. The rate of frequency change is proportional to the adsorbed mass according to the Sauerbrey Equation:

(2) $$\Delta f=\frac{-2f_{0}^{2}\Delta m}{A{\left(\mu \rho \right)}^{\overset{1}{\;}/\underset{2}{\;}}}$$

in which μ is the shear modulus of quartz (2.947 × 10^11^ g·cm^−1^s^−2^), A is the piezoelectrically active crystal area, and ρ is the density of the quartz (2.648 g·cm^−3^). This relation is valid for thin rigid films with uniform mass distribution. Therefore, QCM sensors can be used to measure the mass of various target analytes by immobilizing specific probes on a sensor surface. Bacteriophage probes can be combined with these sensors for specific detection of bacteria. Olsen *et al.* showed that the physical adsorption of ∼3 × 10^10^ phages·cm^−2^ on piezoelectric transducer surface provides a sensitive platform for rapid detection of *Salmonella typhimurium*. This phage-immobilized QCM sensor had a low detection limit of 10^2^ cells·mL^−1^ with a wide linear range of 10–10^7^ cells·mL^−1^ and a rapid response time of less than 180 s [74].

#### 3.2.2. Phage Immobilized Magnetoelastic Sensors

Magnetoelastic sensors oscillate mechanically when an AC magnetic field is applied. The resonance occurs when the frequency of the applied field equals to the natural frequency of sensors. The fundamental resonant frequency of longitudinal oscillations is given by:

(3) $$f=\sqrt{\frac{E}{\rho (1-\sigma^{2})}\frac{1}{2L}}$$

where E is the Young's modulus of elasticity, ρ the density of the sensor material, σ the Poisson's ratio, and L is the long dimension of the sensor. The addition of non-magnetoelastic material to the sensor surface dampens the mechanical oscillation shifting the resonance frequency to lower values:

(4) $$\Delta f=-\frac{f}{2}\frac{\Delta m}{M}$$

where f is the initial resonance frequency, M the initial mass, Δm (smaller than M) the mass change and Δf is the shift in the resonant frequency of the sensor. The response of the magnetoelastic sensors can be measured in the absence of direct physical wire contacts to the sensor, making the possibility of real time and *in vivo* bio-detection systems possible.

Magnetoelastic sensors have been immobilized with filamentous bacteriophages for the detection of various bacteria including *Salmonella typhimurium* and *Bacillus anthracis* spores in different food matrixes such as fat free milk, and fresh tomato [24,67,75]. The limit of detection was typically in the range of 10^3^ cfu·mL^−1^.

### 3.3. Electrochemical Biosensors

#### 3.3.1. Amperometric Biosensors

Amperometry is the most common electrochemical detection method for pathogen detection, and offers better sensitivity compared to other methods. Amperometric biosensors are composed of a reference electrode and a working electrode. A bias voltage is applied to these electrodes to produce a current in the analyte. The current produced directly depends on the rate of electron transfer, which changes with variation in ionic concentration of analyte. Amperometry detects ions in solution by measuring the changes in electric current. Many researched have reported amperometric detection of foodborne pathogens. Neufeld *et al.* combined amperometric technique with phage typing for specific detection of *E. coli* K12, *Mycrobacterium smegmatis*, and *Bacillus cereous* bacteria [77]. These sensors work based on the principle that the phage infection results in bacteria lysis leading to release of bacteria cell content, such as enzyme, into the surrounding medium. This enzymatic activity can be measured and quantified using specific substrate. The product of the reaction between substrate and enzyme is oxidized at the carbon anode at the reference electrode, producing a current. They could achieve limit of detection of 1 cfu·mL^−1^ within 6–8 h using this technique in combination with filtration and pre-incubation before infecting bacteria with phage.

#### 3.3.2. Impedimetric Biosensors

Electrochemicial impedance spectroscopy (EIS) biosensors measure the changes in impedance over a range of frequencies that occur as a result of biomolecular interaction. EIS biosensors have been exploited for bacterial detection by monitoring the changes in the solution-electrode interface due to the capture of microorganisms on the sensor surface. The capture of target analyte such as bacteria on sensor usually increases the impedance due to the insulating properties. Bacteriophages have been used as a crosslinkage between bacteria and electrode surface. For example, Shabani *et al.* showed successful detection of *E. coli* bacteria by immobilizing T4 phage onto the functionalized screen-printed carbon electrode with limit of detection of approximately 10^4^ cfu·mL^−1^ [31]. They observed a decrease in impedance by increasing the bacteria concentration, which is contrary to normal attachment of intact cells on EIS sensor. The reason behind this observation was due to the lytic activity of phages that led to release of ionic intercellular content and increase in conductivity. The specificity of detection was confirmed by using Salmonella as negative control. In a further attempt, Mejri *et al.* observed two successive opposite trends over time for detection of bacteria on phage-EIS biosensors. The impedance initially increased due to the capture of bacteria followed by an impedance decrease attributed to phage-induced lysis. Such dual signals are inclusive to the specific detection of bacteria, and is easily distinguishable from those caused by non-specific binding. They specifically detected *E. coli* with the limit of detection of 10^4^ cfu·mL^−1^ [82]. Although EIS offers label-free detection of pathogens compared to amperometry technique, its application for pathogen detection is limited due to its lower detection limit compared to other techniques.

## 4. Summary

The review critically summarizes various molecular probes that have been exploited for surface functionalization of biosensor platform for pathogen detection. Antibodies and nucleic acid probes have been most extensively used for such applications. However, these probes suffer from several drawbacks including susceptibility to environmental conditions, cross-reactivity, cost-ineffectivity and need of technical expertise. Bacteriophages have recently been looked upon as an attractive alternative probe for pathogen detection owing to their excellent specificity and selectivity to their host and ease of amplification. Initial attempts to realize phage-based sensor platforms relied on their physical adsorption to the surface, but some recent efforts have been made to anchor them using more stable chemical linkages that improves the performance of the sensor platform. Advancement in the genomic information and molecular biology methods also led to the use of genetically engineered phages in the form of phage display technology and reporter phages to tailor the surface property of phages for oriented immobilization and subsequent improvement in pathogen detection. However, these methods are cumbersome and still suffer from whole phage related limitations such as induction of host cell lysis, drying effect resulting in loss of host pathogen capturing ability and limited knowledge of surface functionalization methodology.

The last few years have seen the development of phage RBPs based methods for pathogen detection. The phages RBPs are identified, cloned, expressed, purified and are subsequently functionalized on the sensor platforms for successful detection. Phage RBPs have several advantages over antibody-, nucleic- or whole phage-based probes. They show better endurance to variation in pH and temperature, resistance to proteolytic activity and can be tailored for oriented chemical functionalization on the biosensor surface. Genetic engineering methods can be easily exploited to modify the binding characteristics of the RBPs and knock out enzymatic activity shown by intact phages, which affect the host cell capturing ability. P22 and Sf_6_ phage by e.g., showing endorhamnosidase enzymatic activity, which severely influences the bacterial cell capturing ability of these whole phages as well as wild-type phage RBPs while the RBPs without the enzymatic activity show a relative 2 to 4-fold improvement in host cell capture density [36,62]. RBPs therefore show tremendous promise as molecular probes for specific and selective detection of target pathogens in food and water samples.

The final sections of the review focus on various attempts towards exploiting the phage-based technology for pathogen detection using different biosensor platforms. Intact phages, genetically modified phages as well as recently phage RBPs have been equally explored for pathogen detection in various food samples including meat, dairy products and other produces. Some latest reports on phage RBPs as molecular probes have demonstrated the capability to selectively identify and detect the three most crucial food-borne pathogens namely *Salmonella*, *Campylobacter* and *Shigella*. With the further development of phage RBPs against other economically relevant pathogen and improvement in the detection limits of existing biosensor platforms, the possibility of detecting a single bacterium in a food sample in a high throughput manner would not be impossible.

## 5. Future Outlook

Even though the phage-based pathogen detection technology has come a long way, there are several key issues that are yet to be resolved. Phage RBPs have shown a lot of promise in developing smart and advanced biosensors due to their better stability and ease of integration in a wide variety of detection platforms. However, a concrete method to identify the gene encoding for the RBP in a random phage is not yet established. The existing methods rely on information of the whole genome of a phage to be able to identify, clone, express and characterize a phage RBP, which is an expensive and time-consuming approach. A generic approach therefore has to be developed to achieve the desired. Besides, phages are often considered to be too specific to their host (up to serotype level) with a very narrow range of their potential target, which limits their application in developing pathogen biosensor. While host/target selectivity is highly desirable from the biosensor perspective, it would imply a need for several different recognition elements on the same detection platform to be able to identify all pathogenic serotype of bacteria. Imparting multivalency to the phage RBPs becomes pertinent to design a multiplexed system for simultaneous detection of potent pathogen, an area that is yet to be developed.

The ability to functionalize phages and phage RBPs on metal-based surfaces by physical absorption or chemical anchoring is well established, but a generalized method for their stable anchoring on other sensor surfaces is yet another area that requires significant development. The current state of the art biosensor systems for monitoring food-borne pathogens show a detection limit in the range of 10–10^2^ cfu·mL^−1^ (Table 3), but require extensive sample processing to realize such level of sensitivity. Micro- and nano-cantilever and devices have demonstrated tremendous mass sensitivity up to atto-gram level [83], which can significantly improve the limit of detection. These devices however are fabricated on silicon-based surfaces for which a reliable surface functionalization strategy for phages as well as phage RBPs is yet to be realized. Some preliminary results on the immobilization of His_6_-tagged P22 tail-spike proteins have been demonstrated (unpublished data) but an optimized method is still far from reality. Even though the development of phage and phage RBPs-based detection systems for monitoring food-borne pathogen is still in its infancy, remarkable developments have been made recently and the future of this approach looks promising and bright.

## Figures and Tables

**Table 1.** A list of commercially available nucleic acid-based biosensors for pathogen detection with their mode of detection and the sample source.

**Organism**	**Company**	**Product Name**	**Detection Method**	**Sample Source**
***Candida sp***	Beckton Dickinson, Inc.	BD Affirm™ APIII	DNA hybridization	Vaginal swab
***Chlamydia trachomatis***	Qiagen	HC2 CT-ID	Chemiluminiscence	Endocervical swab
	Gen-probe	APTIMA^®^ CT	TMA */16S RNA	Urine/Urethral swab
	Gen-probe	PACE2 CT	HPA **	Endocervical swab
	Beckton Dickinson, Inc.	BD ProbeTec™ CT	SDA ***	Endocervical swab
	Roche	COBASAMPLICOR CT	PCR	Endocervical/Urethral swab
***Escherichia coli O157:H7***	Qualicon, Inc.	BAX system	Real-time PCR	Water
***Gardnerella***	Beckton Dickinson, Inc.	BD Affirm™ APIII	DNA hybridization	Vaginal swab
***Mycobacterium avium***	Accuprobe^®^	Gen-probe	TMA/RNA	Culture
***Mycobacterium gordonae***	Accuprobe^®^	Gen-probe	TMA/RNA	Culture
***Mycobacterium intracellulare***	Accuprobe^®^	Gen-probe	TMA/RNA	Culture
***Mycobacterium kansasii***	Accuprobe^®^	Gen-probe	TMA/RNA	Culture
***Mycobacterium tuberculosis***	Accuprobe^®^ MTD	Gen-probe	TMA	Sputum
	BD ProbeTec™ ET	Beckton Dickinson, Inc.	SDA	Respiratory andNon-respiratory
	COBAS AMPLICOR MTB	Roche	PCR	Respiratory andNon-respiratory
***Neisseria gonorrhoeae***	Qiagen	HC2 GC-ID	Chemiluminiscence	Endocervical swab
	Gen-probe	APTIMA^®^ GC	TMA/16S RNA	Urine/Urethral swab
	Gen-probe	PACE2 GC	HPA	Endocervical swab
	Beckton Dickinson, Inc.	BD ProbeTec™ GC	SDA	Endocervical swab
	Roche	COBAS AMPLICOR NG	PCR	Endocervical/Urethral swab
***Streptococci*Group A**	Gen-probe	GASDirect^®^	HPA	Pharyngeal swab
***Streptococci*Group B**	Infectio Diagnostic Inc.	IDI-StrepB	Real-time PCR	Vaginal swab
***Trichomonas vaginalis***	Gen-probe	APTIMA^®^	TMA/16S RNA	Urine/ Vaginal swab
	Beckton Dickinson, Inc.	BD Affirm™ APIII	DNA hybridization	Vaginal swab

* Transcription mediated amplification (TMA);

** Hybridization probe assay (HBA);

*** Strand displacement amplification (SDA).

**Table 2.** A list of nucleic acid and protein-based commercial products for foodborne pathogen detection with their method and limit of detection.

**Organism**	**Product**	**Company**	**Method of detection**	**Limit of detection (cfu·mL^−1^)**
***E. coli* O157:H7**	BAX^®^	Dupont	DNA Hybridization	10^4^
	Lateral Flow System	Dupont	Immunoassay	1 (per 25 g food)
	Reveal^®^	Neogen	Immunoassay	10^4^
	GeneQuence^®^	Neogen	Enzyme based	1 (per 25 g food)
	VIDAS	Biomėrieux	Immunoassay	-

***Campylobacter***	BAX^®^	Dupont	DNA Hybridization	10^4^
	VIDAS	Biomėrieux	Immunoassay	-
	ACCUPROBE	Biomėrieux	DNA Hybridization	-

***Listeria***	BAX^®^	Dupont	DNA Hybridization	10^4^
	Lateral Flow System	Dupont	Immunoassay	1 (per 25 g food)
	Reveal^®^	Neogen	Immunoassay	10^6^
	ANSR™	Neogen	DNA Hybridiztion	10^4^
	VIDAS	Biomėrieux	Immunoassay	-

***Salmonella***	ANSR™	Neogen	DNA Hybridiztion	10^4^
	GeneQuence^®^	Neogen	Enzyme based	1 (per 25 g food)
	Reveal^®^	Neogen	Immunoassay	10^6^
	BAX^®^	Dupont	DNA Hybridization	10^4^
	Lateral Flow System	Dupont	Immunoassay	1–4 (per 25 g food)

***Enterobacter***	BAX^®^	Dupont	DNA Hybridization	-
	VIDAS	Biomėrieux	Immunoassay	-

***Vibrio***	BAX^®^	Dupont	DNA Hybridization	10^4^

**Table 3.** A list of phage-based molecular probes exploited for pathogen detection highlighting the transduction platform used and limit of detection achieved.

**Transducer**	**Organism**	**Bioreceptor**	**Limit of detection**	**Ref.**
SPR	*E. coli* K12	T4 Phage	7 × 10^2^ cfu·mL^−1^	[28]
SPR	*E. coli* O157:H7	T4 Phage	10^3^ cfu·mL^−1^	[69]
SPR	MRSA	BP14 Phage	10^3^ cfu·mL^−1^	[69]
SPR	*Salmonella*	P22 Phage TSP	10^3^ cfu·mL^−1^	[36]
SPR	*C. jejuni*	Phage NCTC 12673 TSP	10^2^ cfu·mL^−1^	[64]
SPR	*S. aureus*	Lytic phage (phage 12600)	10^4^ cfu·mL^−1^	[22]
Bioluminesence	*E. coli*	*E. coli* phage	10^3^ cfu·mL^−1^	[70]
Bioluminesence	*Salmonella newport*	Felix phage or Newport phage	10^3^ cfu·mL^−1^	[70]
Bioluminesence	*Salmonella enteritidis*	phage SJ2	10^3^ cfu·mL^−1^	[71]
Bioluminesence	*E. coli* G2-2	AT20	10^3^ cfu·mL^−1^	[71]
Fluorescent	*Staphylococcal enterotoxin B* (SEB)	phage-displayed peptides	1.4 ng	[72]
Fluorescent	*E. coli*	QD-labeled lambda phage	N/A	[73]
Fluorescent	*E. coli*	T7 phage	20 cell·mL^−1^	[68]
QCM	*Salmonella typhimurium*	Filamentous phage	10^2^ cell·mL^−1^	[74]
Magnetoelastic sensors	*Salmonella typhimurium*	Filamentous E2 phage	5 × 10^2^ cfu·mL^−1^	[24,67]
Magnetoelastic sensors	*Bacillus anthracis* spores	Filamentous phage, clone JRB7	N/A	[75]
Amperometric	*Bacillus cereus*	B1-7064 Phage	10 cfu·mL^−1^	[76]
Amperometric	*Mycobacterium smegmatis*	D29 Phage	10 cfu·mL^−1^	[76]
Amperometric combined with pre-filtration	*E. coli* K12	Phage lambda	1 cfu·100mL^−1^	[77]
Impedimetric	*E. coli*	T4 Phage	10^4^ cfu·mL^−1^	[31,78]
